# Renal cyst mimicking hydronephrosis after uterine artery ligation for postpartum haemorrhage

**DOI:** 10.4314/ahs.v22i2.78

**Published:** 2022-06

**Authors:** Nnabuike Chibuoke Ngene

**Affiliations:** 1 Department of Obstetrics and Gynaecology, School of Clinical Medicine, Faculty of Health Sciences, University of the Witwatersrand, Johannesburg, South Africa; 2 Department of Obstetrics and Gynaecology, Leratong Hospital, Krugersdorp, Gauteng, South Africa

**Keywords:** Uterine artery ligation, preliminary imaging report, ureteric injury

## Abstract

**Background:**

The proximity of the uterus and the cervix to the urinary tract predisposes the latter to injury during obstetrical and gynaecological surgical procedures. Following a difficult surgical procedure on the lower uterine segment and or adnexa, urinary tract injury should be excluded.

**Methods:**

A booked 39-year-old G3P2 lady who suffered an ischaemic stroke in the index pregnancy had a caesarean delivery at 39 weeks of gestation and sustained an extensive tear that extended inferiorly on the left lateral aspect of the uterus and this resulted in postpartum haemorrhage. Following the repair of the tear, uterine artery ligation was performed to achieve haemostasis.

**Results:**

Postoperatively, conventional ultrasonography which was performed to exclude ureteric injury suggested left hydronephrosis and a preliminary report of computerized tomography (CT) showed the same finding. The patient subsequently had left ureteric stenting. The final report of the CT scan was delayed but showed a simple left renal cyst and no hydronephrosis.

**Conclusion:**

Renal cyst is a differential diagnosis of hydronephrosis. Delayed availability of the final result of medical investigations jeopardises patients' safety. A preliminary imaging report is prone to error and its use to determine the indication for an invasive procedure should be limited to emergencies.

## Background

One of the measures to control postpartum haemorrhage (PPH) is uterine artery ligation.^1^–^4^ The procedure is indicated when haemostatic measures applied at a site of bleeding from the uterus are ineffective, when suturing tear involving the uterine artery, and as part of a step during sequential devascularization for uterine bleeding. Details of the surgical procedure are described latter. However, the procedure may be complicated by ureteric injury which is preferably managed or excluded during the same operative session. Some clinicians perform imaging such as abdominopelvic ultrasonography postoperatively to exclude ureteric injury particularly if the uterine arteries are ligated at a level considered to be unusually low on the lower uterine segment. Prompt availability of the final imaging report is important for making appropriate decision and preventing unnecessary additional intervention.

In the case presented, a woman living with HIV had a stroke during pregnancy. She had caesarean delivery at term and developed PPH which was controlled with uterine artery ligation. Postoperatively, ultrasonography and preliminary report of computerized tomography (CT) intravenous pyelogram (IVP) misdiagnosed renal cyst as hydronephrosis and the patient had stenting of left ureter before the final CT report with the correct diagnosis was available. This report discusses the following lessons: (i) delay in availability of medical investigation report which frequently occurs in low-resource settings, (ii) succinct technique of uterine artery ligation and how to prevent and evaluate inadvertent ligation of the ureter, and (iii) adjunct obstetric concerns. This article is the first reported case of renal cyst mimicking hydronephrosis in the context of PPH amenable to uterine artery ligation.

## Case presentation

In details, a 39-year-old G3P2 lady commenced antenatal care in a private hospital. She was a woman living with HIV and had been on treatment since 2012 with a CD4 count of 399 cells/µl. The viral load was lower than detectable. The patient was obese (body mass index 41.85 kg/m^2^) with a normal obstetric ultrasound and blood pressure of 121/80 mmHg during the first antenatal clinic visit. She suffered ischaemic stroke at 20 gestational weeks and developed slurred speech and left hemiparesis with limping gait (power on the left limbs was 4/5).

The patient was referred to a tertiary hospital where she was investigated by a multidisciplinary team including a neurologist. The results of special investigations for stroke^5^ including magnetic resonance imaging of the brain and thrombotic screening were normal. The patient had no thrombolysis because she was pregnant and presented > 4.5 hours of stroke onset. She was commenced on enoxaparin and physiotherapy. At 34 gestational weeks, the patient developed mild pre-eclampsia which was investigated and controlled with methyldopa and the pregnancy progressed to term. After counselling on the appropriate route of delivery, she had an elective caesarean delivery based on her preference at 39 gestational weeks. The caesarean delivery was performed under general anaesthesia. The pre-operative haemoglobin was 10.5 g/dl. The enoxaparin was omitted 24 hours before the delivery. A normal female baby was delivered with a birth weight of 2825 g, and Apgar scores of 6 and 9 in 1 and 5 minutes respectively. The patient sustained a tear that extended the uterine incision on the left lateral aspect of the uterus. She developed PPH and a specialist obstetrician was invited and participated in the intra-operative management. Other causes of PPH such as uterine atony, retained products of conception and coagulopathy were excluded. The bladder was mobilised inferiorly and the uterine incision and tear were repaired. Bilateral uterine artery ligation was performed to achieve haemostasis. The course of the ureters was identified but bleeding made it difficult to completely exclude ureteric injury. She lost 1000 ml of blood and the haemoglobin concentration in the postoperative period was 9.3 g/dl.

**Figure 1:**
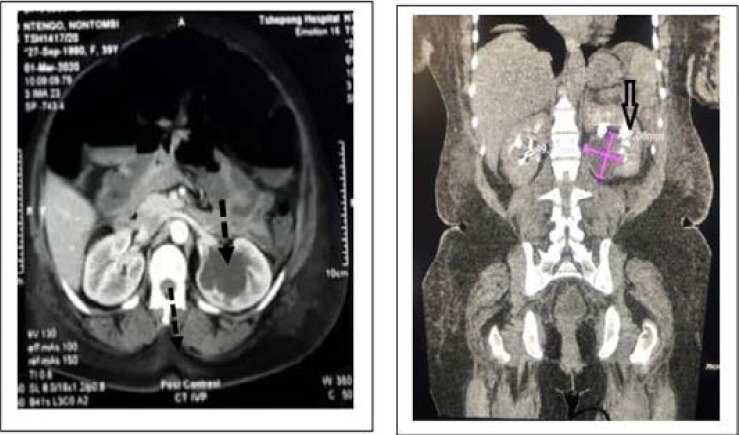
Computerized tomography showing left renal cyst (dash-type arrow), and collecting systems filled with contrast (block-type arrow) which are distinct from the renal cyst measuring 49.71 X 57.08 mm.

A day after delivery (day 1 postpartum), abdominopelvic ultrasonography was requested based on the local protocol (unpublished) to exclude intra-operative ureteric injury. The ultrasonography was performed on day 2 postpartum and showed features of left hydronephrosis. The renal function test was normal. CT IVP was requested on day 3 postpartum. The facility for CT IVP was unavailable at the base hospital and the imaging was performed in another health facility on day 4 postpartum due to transport challenges. The preliminary report of the CT IVP suggested features of hydronephrosis. Following a 24-hour delay without receiving the final CT IVP report, retrograde ureterogram was performed on day 5 postpartum and was normal. At the same time, a stent was inserted in the left ureter because of the possibility that the ureter could be kinked. The final report of CT IVP was obtained 12 hours after the stent had been inserted and showed simple left renal cortical cyst (Bosniak I lesion) 6 with no hydronephrosis (Figure 1) and required no further treatment.^7^ She was counselled, discharged home with her baby in a stable condition and planned for stent removal 6 weeks post-insertion. The patient understood that there is maximum stent life, and that some of the possible complications of delay in removal of forgotten indwelling ureteric stents include severe urinary tract infection, stent fragmentation, stent migration, encrustation and stone formation which may result in obstructive uropathy, hydronephrosis, renal impairment and death.^8^–^10^ Unfortunately, she was lost to follow-up despite different attempts to contact her.

## Discussion

The index case report shows that the scope of routine antenatal ultrasonography in the early pregnancy is limited^11^ and does not include assessment of the maternal renal system.^12^ As highlighted in Table 1, the index report demonstrates the need to consider the assessment of maternal renal system during the first trimester ultrasonography. It is concerning that the result of the abdominal ultrasonography which was performed during investigation of the stroke showed normal renal system and did not comment on any cyst.

**Table 1:** Highlights

S/No	
1	First-trimester ultrasonography should include assessment of renal calyces where possible, to prevent misdiagnosis of a pre-existing renal lesion as a complication of childbirth.
2	Renal cyst is a differential diagnosis of hydronephrosis.
3	Caution should be exercised when deciding on a surgical intervention based on a preliminary imaging report.
4	Health facilities, particularly those in low-resource settings, should strive to develop and implement measures to prevent delay in performing medical investigations and should also ensure that the final reports of results of these investigations are available to the healthcare professionals timeously.

Furthermore, the delay in receiving the final CT IVP report was a major concern. This is a challenge associated with some medical investigations requested in many low-resource settings. The delay may be caused by reasons such as lack of onsite capacity, power outage, lack and faulty equipment, delay in payment of medical services by patients,^13^ transport difficulty and unavailability of qualified health care professional to report the investigation. Health facilities, particularly those in low-resource settings, should strive to address the specific challenges that delay investigations/reports. In the index case, the one-man specialist radiologist was not available and this caused the delay. Employment of an additional radiologist is a possible measure that may prevent this type of delay.

*To perform uterine artery ligation*, the bladder is mobilized inferiorly^14^ below the site of bleeding, and an abdominal swab is placed behind the uterus to protect the intestine. A large needle with an absorbable suture^1^ is passed from anterior to posterior surfaces of the uterus 2 – 3 cm medial to the ipsilateral side of the uterus. The same suture is pierced through an avascular area in the broad ligament from posterior to anterior, and the encircled tissues and vessels are ligated. Mobilizing the bladder inferiorly and ensuring that the suture which is passed through the broad ligament remains close to the uterus as practically as possible prevents ureteric injury. When the uterine arteries are ligated at the lowermost part of the uterus, the risk of ureteric injury increases. After achieving haemostasis in a stable patient, it is prudent to trace the course of the ureter in the ipsilateral side to ensure that it is not ligated. In an unstable patient, tracing the ureters will increase the duration of the surgery and may provoke bleeding. In an unstable patient, therefore, it is preferable to insert an abdominal drain, complete the surgery, monitor physiologic indices and perform abdominopelvic ultrasonography to exclude ureteric injury as soon as the patient is stable. In our centre, we perform abdominopelvic ultrasonography postoperatively to exclude ureteric injury if the uterine arteries are ligated at an unusually low level. This was the situation in the case presented. CT IVP, also called urogram, is the preferred less invasive modality for further evaluation of the presence or absence of ureteric injury.^15^ As shown in the index case report, a renal cyst may mimic hydronephrosis and this informs the superiority of CT IVP over conventional ultrasonography. Contrast-enhanced ultrasonography is also superior to conventional ultrasound examination in diagnosing renal cyst.^6^ The differentiating feature is that renal cyst does not have any connecting channel with the calyceal collecting duct.^16^ Unfortunately, the healthcare professional who provided the provisional report of CT IVP was inexperienced. Another recommended method of diagnosing hydronephrosis is retrograde ureterogram but this is invasive and involves cystoscopy and taking multiple X-rays using a C-arm to asses flow of contrast. In the index case, the flow of contrast in both ureters was satisfactory but the attending urologist decided to stent the left ureter as it could be kinked given the available imaging reports.

## Conclusion

clinicians should consider renal cyst as a differential diagnosis of hydronephrosis and exercise caution when deciding on a surgical intervention based on preliminary imaging report. Assessment of renal calyces during antenatal ultrasonography, where possible, may prevent misdiagnosis of a pre-existing renal lesion as a complication of childbirth.
